# The spatial effects of rural toilet retrofitting investment on farmers' medical and health expenditure in China

**DOI:** 10.3389/fpubh.2023.1135362

**Published:** 2023-06-23

**Authors:** Xin Zheng, Fu-Xia Yang, Dong-Shou Fan, Zao-Ning Yang

**Affiliations:** ^1^College of Economics and Management, Huazhong Agricultural University, Wuhan, Hubei, China; ^2^School of Materials Science and Engineering, Xi'an Polytechnic University, Xi'an, Shaanxi, China

**Keywords:** rural toilet retrofitting investment, farmers' medical and health expenditure, spatial effect, spatial heterogeneity, spatial correlation

## Abstract

**Background:**

China stretches across a vast area, and different geographical environments and economic and social development conditions, along with learning imitation and factor flow among participants can lead to two major spatial characteristics of toilet retrofitting investment: spatial heterogeneity and spatial correlation.

**Methods:**

This study contributes to explore this topic by assessing the spatial heterogeneity and spatial correlation of toilet retrofitting investment on farmers' medical and health expenditure based on the spatial econometric model.

**Results:**

(1) There are significant spatial agglomeration characteristics of both the toilet retrofitting investment and farmers' medical and health expenditure in China. (2) At the national level, the rural toilet retrofitting investment will influence the farmers' medical and health expenditure, and the effect on the local area is greater than on the surrounding areas. (3) After taking into account the differences in natural geographical environment and social and economic development, China is divided into four regions: east, central, west and northeast. In terms of spatial effects within different regions, the intensity of the impact of toilet retrofitting investment on local farmers' medical and health expenditure is in the order of central > eastern > western > northeast. The improvement of people's livelihood in the eastern and central regions by toilet retrofitting investment would lead to imitation by surrounding regions, thus reflecting spillover effects, while in the western region, toilet retrofitting investment would trigger fierce competition in related industries and factor markets, manifesting the competition effect. (4) As for the spatial effects across different regions, the toilet retrofitting investment produces spillover effects in all four regions, among which the intensity of the influence effect is the greatest in the central-western region, followed by the west-northeast, and the influence effect in the east-west is not significant.

**Discussion:**

The comprehensive promotion of rural toilet retrofitting should not only focus on investment in the western and northeastern regions, but also strengthen regional communication and cooperation to improve rural residents' health and quality of life.

## 1. Introduction

Improving citizens' health can influence people's wellbeing and economic development by improving human capital (1). With the successful completion of building a moderately prosperous society and the complete victory of the battle against poverty in 2021, health problems caused by food shortages and nutritional deficiencies have basically disappeared, and the physical health of rural residents' is more constrained by excessive nutrition and poor sanitation conditions (2). However, compared with the rapid change of the urban landscape, the rural development is relatively slow, especially under multiple constraints of economy, system and concept, thus the problem of rural residential environment is increasingly prominent (3). Thereinto, traditional dry toilets not only provide a suitable environment for the propagation of the mosquitoes and flies, and the breeding of the pathogenic microorganisms and parasites, but also cause the infiltration of untreated feces into natural water bodies to pollute water sources, leading to the spread of diseases, which has seriously threatened the health of hundreds of millions of rural residents' (4). For this reason, the Chinese government has attached great importance to toilet retrofitting work, and has successively implemented a series of supporting policies and continuously increased investment in rural toilet retrofitting. By 2020, the rural toilet retrofitting investment in China has reached 180.392 billion yuan, and the penetration rate of sanitary toilets nationwide has increased to 68%, achieving a milestone achievement^①^. However, China stretches across a vast area, and different geographical environments and economic and social development conditions, along with learning imitation and factor flow among participants can lead to two major spatial characteristics of toilet retrofitting investment: spatial heterogeneity and spatial correlation. Therefore, it is of great practical significance to clarify the spatial characteristics of the impact of toilet retrofitting investment on farmers' medical and health expenditure, and to explore how to adjust investment strategies in a targeted manner for the implementation of the Healthy China Initiative and the Rural Revitalization Strategy.

Increasing toilet retrofitting investment is an effective way to improve the health of rural residents. It is generally acknowledged that an increase in toilet retrofitting investment will accelerate the process of local dry toilet retrofitting and improve the health of rural residents' by reducing disease transmission and controlling environmental pollution effectively (4), thereby reducing expenditure on medical and health. In other words, areas with high toilet retrofitting investment are expected to have lower medical and health expenditure, and vice versa. However, relevant statistics indicate that farmers' medical and health expenditure is increasing in most provinces of western and northeastern China, despite the fact that the average annual growth rate of investment in toilet retrofitting has reached 2.33% and 2.12% in these regions^①^. In response to the phenomenon of expectation and reality contradictory, some studies have revealed that the agglomeration of factors caused by the increasing investment and the spatial dependence of public health care levels are important reasons for this phenomenon (5, 6). Although some scholars have begun to emphasize the heterogeneity of rural toilet retrofitting in different regions, in addition to heterogeneity, the toilet retrofitting investment and its effects are also spatially correlated. As a matter of fact, the natural geography environment and economic and social development vary greatly among various regions in China, thus the investment efficiency of rural toilet retrofitting will inevitably lead to unbalanced distribution that is higher in the east and lower in the west (7), which in turn will lead to spatial differences in the toilet retrofitting investment and its effect, resulting in differences in farmers' medical and health expenditure in different regions. Moreover, as a systematic public project, rural toilet retrofitting requires not only government guidance and public participation, but also market-oriented operation and social support, and inter-subject learning imitation and factor clustering in a certain region, are important reasons for the spatial correlation (8, 9). Therefore, when exploring the spatial effects of investment-driven farmers' physical health improvement, we should not only consider spatial heterogeneity, but also take into account spatial correlation. This provokes the following questions: After considering spatial factors, is there spatial heterogeneity and spatial correlation in the impact of toilet retrofitting investment on farmers' health care expenditure? Are there differences in spatial heterogeneity and spatial correlation among different regions? According to the spatial characteristics, how can investment strategies be optimized to reduce farmers' health care expenditure? Therefore, this paper focuses on exploring the spatial heterogeneity and spatial correlation of rural toilet retrofitting investment affecting farmers' health care expenditure, and compares the spatial effects within and among different regions, so as to provide a scientific basis for national investment strategy adjustment, and improvement of people's livelihood.

## 2. Literature review and theoretical analysis

### 2.1. Toilet retrofitting investment and farmers' medical and health expenditure

The existing literature on the impact of toilet retrofitting investment on farmers' medical and health expenditure mainly discusses the three aspects including: disease reduction, prevention of environmental pollution and improvement of human capital. First of all, in terms of disease reduction, some studies have found that investments could effectively promote rural dry toilet retrofitting, reduce the environment suitable for the breeding of disease-causing microorganisms and mosquitoes and flies, and limit the incidence of diseases such as malaria and diarrhea by reducing the transmission of viruses and pathogens such as E. coli and Salmonella, and the breeding of parasites such as helminths and schistosomes (10–12), to protect the health of rural residents to reduce their medical and health expenditures.

Secondly, to prevent and control environmental pollution, some scholars believe that the toilet retrofitting investment can effectively prevent pathogenic microorganisms and parasitic eggs from entering natural water bodies and causing water pollution by accelerating the construction of sanitary toilets in rural areas, and block the transmission of water-borne infectious diseases and other related diseases (13), thus reducing the farmers' medical and health expenditure. Finally, in terms of improving human capital, toilet retrofitting investment promotes the popularization of sanitary toilets in rural areas, and it is helpful to improve the health human capital of rural residents' (14) to reduce their medical and health expenditure. Based on this, the following hypothesis is proposed:

Hypothesis 1: Toilet retrofitting investment can affect farmers' medical and health expenditure.

### 2.2. The spatial effects of toilet retrofitting investment on farmers' medical and health expenditure

Since the spatial effects mainly include spatial heterogeneity and spatial correlation, the spatial effects of toilet retrofitting investment on farmers' medical and health expenditure are analyzed from these two aspects.

Spatial heterogeneity refers to the non-equilibrium characteristics of the effect of toilet retrofitting investment on farmers' medical and health expenditure at the spatial level. In view of different areas in the natural geographical environment, economic and social development and other aspects, there are significant differences in the demand for toilet retrofitting in different regions, which leads different effects of rural toilet retrofitting in different regions, resulting in different health care expenditures of farmers in different regions. Specifically, on the one hand, China's terrain exhibits a stepped pattern with higher elevation in the west and lower elevation in the east, and there are significant differences in climate, altitude and location. Water resources gradually decrease from the southeast coast to the northwest inland (15), resulting in very different costs for toilet retrofitting in different regions (16). On the other hand, the socio-economic development in the eastern, central and western region is unbalanced, and the gap between coastal and inland is large in terms of economic development, and the residents in remote areas and developed areas differ greatly in living customs and traditional concepts (17), which also lead to different demands for toilet retrofitting in different regions, thus affecting farmers' medical and health expenditure. At present, there are abundant literature on the spatial heterogeneity of investment in of farmers' medical and health expenditure affected by toilet retrofitting investment, and a large number of studies have found that the differences in natural geographic environment (18), economic development level (5, 19), rural public services (20), social security system (21, 22), community development (23), local customs (24), traditional concepts (25), and health awareness (26) will lead to the significant differences in the effect of toilet retrofitting investment on farmers' medical and health expenditure in different regions, resulting in spatial heterogeneity characteristics in farmers' medical and health expenditure.

Spatial correlation refers to the “spillover effect” or “competitive effect” caused by the change in toilet retrofitting investment in a certain area, which influences the farmers' medical and health expenditure in surrounding areas. Although some scholars have qualitatively analyzed the spatial correlation of toilet retrofitting investment and its effects, and the exploration of the successful cases brought by toilet retrofitting investment will be imitated and learned by other regions (27), quantitative empirical studies are not yet available. The current literature mainly explains spatial correlation in terms of inter-subject learning and factor market competition among participating subjects. From the perspective of inter-subject imitation and learning, on the one hand, for their own development, local government will imitate and strategically interact with each other in terms of fiscal expenditure, industrial structure, institutional structure (28). Moreover, the Chinese-style “top-down” scale competition will lead local officials to learn from the advanced experience of better performing areas for political achievements, and this cross-regional institutional imitation will help promote the rural toilet retrofitting work, improve the health of farmers in surrounding areas, and then reduce their medical and health expenditure. On the other hand, in the process of rural toilet retrofitting, there are product supply, project bidding, planning and design and construction, and other links. The enterprises will apply the advantages of products, technology patents, advanced technology and management mode and other aspects of knowledge to the toilet retrofit project to reduce costs. In turn, frequent communication and cooperation between enterprises enable neighboring enterprises to learn knowledge and technologies to improve their production efficiency and promote rural toilets retrofitting in surrounding areas (29), thereby reducing the farmers' medical and health expenditure in those areas. Similarly, the experience and management model explored by enterprises or communities in maintenance, sewage absorption, and recycling can attract enterprises or communities in surrounding areas to observe and learn through the publicity of “successful examples” and “advanced models,” thereby driving the management and care of toilets in the surrounding areas (27). In other words, enterprises and communities can improve technical level and the quality of products and services through the form of “learning by watching” and “learning by doing” (30) to satisfy the demand for toilet retrofitting in surrounding areas, thus reducing their health care expenditure and generating the “spillover effect.” Unlike the learning and imitating effect, the huge demand for toilet retrofitting investment will also promote the development of related industries and prompt enterprises to increase investment, leading to increased factor demand and fierce competition among enterprises for various resources, such as technology, talent and production materials (31). It will also promote the flow of various factors to areas with faster growth of toilet retrofitting investment. Due to the limited resources, rural toilet retrofitting work in surrounding areas is delayed, and the farmers' health in these areas are difficult to be improved, which in turn increases their medical and health expenditure and generates the “competition effect.”

To sum up, few literature has taken into account both the spatial heterogeneity and spatial correlation in the impact of toilet retrofitting investment on farmers' medical and health expenditure. This paper incorporates both spatial characteristics into the same analytical framework and proposes the following hypothesis:

Hypothesis 2: There are spatial effects in the impact of toilet retrofitting investment on farmers' medical and health expenditure.

### 2.3. The differentiation of spatial effects in different regions

Most of the literature uses traditional econometric models to analyze the heterogeneity of rural toilet retrofitting, and these traditional models are usually based on the premise assumption of spatial homogeneity or spatial independence, which could not better reflect the spatial differences of the impact of toilet retrofitting investment on farmers' medical and health expenditure. The conclusions drawn are also mainly that the impact of toilet retrofitting investment in different regions on farmers' medical and health expenditure shows heterogeneity, but the spatial characteristics within and between different regions are less involved (32). However, according to the analysis above, there should be significant spatial differences in the impact of toilet retrofitting investment on farmers' health care expenditure, and the spatial differences will be reflected by different regional spatial effects (4). Therefore, on the basis of empirical test of the spatial effects of toilet retrofitting investment on farmers' medical and health expenditure, this paper further explores the spatial differences of toilet retrofitting investment affecting farmers' medical and health expenditure, based on which the following hypothesis is proposed:

Hypothesis 3: There are spatial differences in the impact of toilet retrofitting investment on farmers' medical and health expenditure.

## 3. Research design

Considering the spatial correlation between toilet retrofitting investment and farmers' medical and health expenditure, an appropriate econometric model should be selected for empirical test. Cross panel data focuses on the analysis of cross effects among variables in different regions (33, 34). However, this approach cannot identify intra- and inter-regional cross effect. To address this question, the spatial econometric model which can accurately identify intra- and inter-regional spatial effect by adjusting weights is selected in our study to empirically test the health effect of rural toilet retrofitting investment.

### 3.1. Research method

#### 3.1.1. Spatial correlation analysis

Usually, before using the spatial metrology model, it is necessary to determine whether the research object has the spatial correlation. In this paper, the classical method is used to test the spatial correlation between toilet retrofit investment and farmers' medical and health expenditure. The formula is as follows:


(1) $$\begin{array}{l} I\text{=}\frac{n\overset{n}{\underset{i=1}{\sum }}\overset{n}{\underset{j\neq 1}{\sum }}W_{ij}\left(x_{i}-\overset{-}{x}\right)\left(x_{j}-\overset{-}{x}\right)}{\overset{n}{\underset{i=1}{\sum }}\overset{n}{\underset{j\neq 1}{\sum }}W_{ij}\overset{n}{\underset{i=1}{\sum }}{\left(x_{i}-\overset{-}{x}\right)}^{2}} \end{array}$$

In the above equation, *x*_*i*_ and *x*_*j*_ are the values of toilet retrofitting investment (or farmers' medical and health expenditure) at adjacent points; $\overset{-}{x}$ is their average values; *W* is the spatial weight matrix, *W*_*ij*_ is the element of row *i* and column *j* in space weight matrix; and *n* is the number of study areas.

The significance of Moran' I can be tested by *Z* values:


(2) $$\begin{array}{l} Z=\frac{I-E\left(I\right)}{\sqrt{VAR\left(I\right)}} \end{array}$$

In Equation (2), *E*(*I*) and *VAR*(*I*) represent the expectation and variance of Moran' I, respectively. In general, when |*Z*|> 1.96, it means that there is a significant correlation between the research objects. In addition, the Moran scatter plot can also more intuitively present the local correlation characteristics of the research objects.

#### 3.1.2. The measurement of spatial effects

With the help of the Spatial Dubin Model (SDM), this paper explores the influence of toilet retrofitting investment on farmers' medical and health expenditure. According to LeSage et al. (35), the SDM can be expressed as:


(3) $$\begin{array}{l} Y=\rho WY\text{+}\beta X+\theta WX+\xi \end{array}$$

In Equation (3), *Y* is farmers' medical and health expenditure; *WY* is the lag term; *X* is the explanatory variables including toilet retrofit investment, temperature and public service level; *WX* is the lag term of the explanatory variables; ρ, β and θ represent regression coefficients; *W* is the spatial weight matrix; and ξ is the random disturbance term, which is independent of time and regional variables.

First, the appropriate weight matrix should be determined. To ensure the robustness of the empirical results, following the research of Zhong (36), this paper also considers two weight matrices: spatial adjacency matrix and geographical distance matrix. The spatial adjacency weight matrix is based on the binary algorithm. The geographically adjacent regions are assigned the value “1” and the geographically non-adjacent regions are assigned the value “0.” The spatial adjacency matrix is defined as follows:


(4) $$W_{ij}=\{\begin{array}{ll} 1, & i\;and\;j\;is\;adjacent\\ 0, & i\;and\;j\;is\;not\;adjacent \end{array}$$

The geographical distance weight matrix is set by the reciprocal of geographical distance between two provincial capitals *d*_*ij*_; the geographical distance is the linear Euclidean distance between provincial capitals. The closer the distance between two provinces, the greater the weight. The geographical distance matrix is defined as follows:


(5) $$W_{ij}=\{\begin{array}{ll} 1/d_{ij}, & i\neq j\\ 0, & i=j \end{array}$$

In the estimation of the model, the LR test and Wald test are used to determine whether the spatial Durbin model (SDM) can be simplified into a spatial lag model (SLM) or spatial error model (SEM). When $H_{0}^{1}$:θ = 0, SDM can be simplified to SLM, and when $H_{0}^{2}$:θ+ρβ = 0, SDM can be simplified to SEM.

Unlike the traditional linear regression equation, each regression coefficient in the spatial econometric model is no longer the direct reflection of the influence degree of the explanatory variables, but it is reflected by the total, direct and indirect effects. The direct effect refers to the average influence of explanatory variables such as toilet retrofitting investment on the farmers' medical and health expenditure in the region, and the indirect effect refers to the average influence of explanatory variables such as toilet retrofitting investment on the farmers' medical and health expenditure in surrounding regions. The total effect is the average influence of explanatory variables such as toilet retrofitting investment on the farmers' medical and health expenditure in all regions. Thus, the total effect is the sum of the direct and indirect effects. Then Equation (3) can be adjusted as follows:


(6) $$\begin{array}{l} \left(I_{\text{n}}-\rho W\right)Y=\beta X+\theta WX+\xi \end{array}$$

Multiply both sides of Equation (6) by${\left(I_{n}-\rho W\right)}^{-1}$, and expand it as follows:


(7) $$\begin{array}{l} Y=\overset{k}{\underset{r=1}{\sum }}S_{r}\left(W\right)x_{r}-V\left(W\right)\xi \end{array}$$

In the above equation, *S*_*r*_(*W*) = *V*(*W*)(*I*_*n*_β_*r*_+*Wθ*_*r*_), $V\left(W\right)={\left(I_{n}-\rho W\right)}^{-1}$, expanding Equation (7), the following is derived:


(8) $$\begin{array}{l} \left(\begin{array}{c} Y_{1}\\ Y_{2}\\ \vdots \\ Y_{n} \end{array}\right)=\sum_{r=1}^{k}\left(\begin{array}{cccc} S_{r}{\left(W\right)}_{11} & S_{r}{\left(W\right)}_{12} & \cdots & S_{r}{\left(W\right)}_{1n}\\ S_{r}{\left(W\right)}_{21} & S_{r}{\left(W\right)}_{22} & \cdots & S_{r}{\left(W\right)}_{2n}\\ \vdots & \vdots & \ddots & \vdots \\ S_{r}{\left(W\right)}_{n1} & S_{r}{\left(W\right)}_{n2} & \cdots & S_{r}{\left(W\right)}_{nn} \end{array}\right)\\ \;\times \left(\begin{array}{c} X_{1r}\\ X_{2r}\\ \vdots \\ X_{nr} \end{array}\right)+V(W)\xi \end{array}$$

Therefore, the total, direct and indirect effects can be expressed as follows:


(9) $$\begin{array}{l} \overset{-}{M}{\left(r\right)}_{\text{Total effect}}\text{=}n^{-1}l_{n}^{-1}S_{r}\left(W\right)l_{n} \end{array}$$

(10) $$\begin{array}{l} \overset{-}{M}{\left(r\right)}_{\text{Direct effect}}\text{=}n^{-1}tr\left(S_{r}\left(W\right)\right) \end{array}$$

(11) $$\begin{array}{l} \overset{-}{M}{\left(r\right)}_{\text{Indirect effect}}\text{=}\overset{-}{M}{\left(r\right)}_{\text{Total effect}}-\overset{-}{M}{\left(r\right)}_{\text{Direct effect}} \end{array}$$

In Equation (9), (10) and (11), $\overset{-}{M}{\left(r\right)}_{\text{Total effect}}$, $\overset{-}{M}{\left(r\right)}_{\text{Direct effect}}$ and $\overset{-}{M}{\left(r\right)}_{\text{Indirect effect}}$ are expressed as the total, direct and indirect effects, respectively; and $l_{n}={\left(1\cdots 1\right)}_{1\times n}^{T}$.

### 3.2. Variables setting

#### 3.2.1. Explained variable

Farmers' medical and health expenditure (FME). According to the research of Ma et al. (22), it is difficult to properly determine the actual effect of rural toilet retrofitting activities from a simple analysis of farmers' medical and health expenditure. Moreover, when the proportion of medical and health expenditure in income decreases continuously, farmers' welfare becomes better and their medical and health burden gradually reduces. Therefore, the ratio of farmers' per capita medical and health expenditure^②^ to per capita disposable income is used to measure their medical and health expenditure.

#### 3.2.2. Core explanatory variable

Rural toilet retrofitting investment (RTI). It is expressed as the logarithm of rural toilet retrofitting investment.

#### 3.2.3. Other variables

The other variables are defined below. (1) Local public service (lnPS). The logarithm of general fiscal expenditure is used to represent the level of local public services. (2) Rural medical and health care (RH). The number of clinics per thousand rural population is used to represent the differences in rural health care. (3) Rural elderly care facilities (REF). The level of old-age care level in rural areas is measured by the number of nursing homes per thousand rural population. (4) Rural medical insurance (RMI). The coverage rate of new rural cooperative medical insurance is used to represent the rural medical insurance level. (5) Rural minimum living guarantee (lnRLG). The logarithm of rural minimum living guarantee expenditure is used to represent the minimum living guarantee level. (6) Rural per capita consumption (lnRPC). The logarithm of per capita consumption expenditure of rural households is used as the indicator of consumption level. The statistical results of each variable are presented in Table 1.

**Table 1:** The descriptive analysis of variables.

**Variables**	**Variables definition**	**Mean**	**Standard error**	**Maximum**	**Minimum**
*FME*	The ratio of farmers' per capita medical and health expenditure to per capita disposable income	0.067	0.021	0.150	0.025
*lnRTI*	The logarithm of the rural toilet retrofitting investment	0.536	1.305	−6.077	3.609
*lnPS*	The logarithm of local general public fiscal expenditure	5.438	0.690	6.996	3.478
*RH*	Clinic numbers of per 1,000 rural population	0.961	0.333	1.833	0.341
*REF*	Nursing home numbers per 1,000 rural population	0.043	0.028	0.217	0.001
*RMI*	The coverage rate of new rural cooperative medical insurance	0.652	0.184	0.205	0.862
*lnRLG*	The logarithm of rural minimum living guarantee expenditure	2.180	1.400	4.077	−6.659
*lnRPC*	The logarithm of per capita consumption expenditure of rural households	8.474	0.507	9.643	7.395

### 3.3. Data sources

In this paper, 30 Chinese provinces (municipalities or autonomous regions), excluding Hong Kong, Macao, Taiwan, and Tibet Province are studied from 2006 to 2017. The data on the explained variable of farmers' medical and health expenditure come from the China Statistical Yearbook. The data on the core explanatory variable of rural toilet retrofitting investment is derived from the China Environmental Yearbook. The data on other economic and social variables are derived from the China Statistical Yearbook, China Rural Statistical Yearbook and Economy Prediction System (EPS) database. Further, to eliminate the influence of inter-annual price rises and inflation, we take 2006 as the base year and convert the relevant data using the deflator.

## 4. Results and discussion

### 4.1. Spatial correlation test

Table 2 presents the calculation results of the global Moran Index of rural toilet retrofitting investment and farmers' medical and health expenditure from 2006 to 2017. Table 2 indicates that from 2006 to 2017, the global Moran Index of rural toilet retrofitting investment and farmers' medical and health expenditure were significantly positive in most years, and the Z value of the normal statistic of the Moran Index is at the critical value at the significance level of 1%, implying obvious spatial correlation. It suggests that toilet retrofitting investment and farmers' health care expenditure are not randomly distributed, but regions with similar growth rates of medical and health expenditure and toilet retrofitting investment tend to be spatially agglomerated. Thus, both the faster and slower development regions tend to cluster, which has obvious characteristics of the “Matthew effect.” It is consistent with the research result of Liu and Liu (4).

**Table 2:** Global Moran's I index of rural toilet retrofitting investment and farmers' medical and health expenditure.

**Year**	**lnRTI**		**FME**	
	**Moran' I**	**Z Value**	**Moran' I**	**Z Value**
2006	0.197^***^	3.676	0.235^***^	4.267
2007	0.244^***^	4.477	0.268^***^	4.803
2008	0.129^***^	2.610	0.272^***^	4.872
2009	0.222^***^	4.153	0.292^***^	5.162
2010	0.115^***^	2.414	0.243^***^	4.417
2011	0.131^***^	2.663	0.150^***^	3.009
2012	0.116^***^	2.716	0.235^***^	4.334
2013	0.126^***^	2.831	0.239^***^	4.330
2014	0.104^**^	2.243	0.252^***^	4.598
2015	0.076^**^	1.836	0.232^***^	4.529
2016	0.011	0.740	0.263^***^	4.862
2017	0.109^***^	2.318	0.245^***^	4.436

*,** and *** represent the significance level of 10, 5, and 1%, respectively. All figures are rounded results.

**Figure 1:**
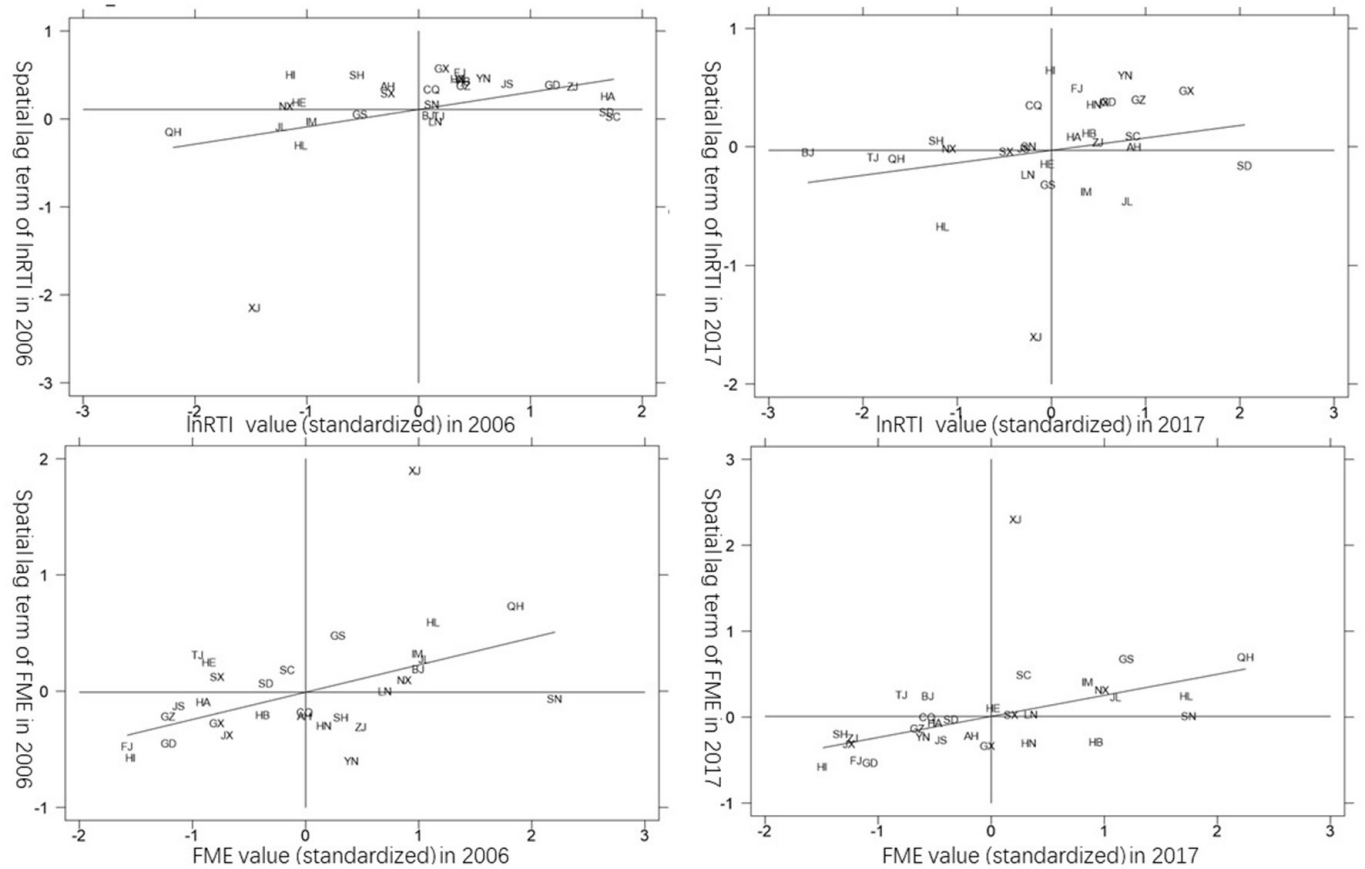
Moran scatter plot of China's rural toilet retrofitting investment and farmers' healthcare expenditure in 2006 and 2017.

This paper plots Moran' I scatter plots of toilet retrofitting investment and farmers' medical and health expenditure in 2006 and 2017 (Figure 1)^③^. It can be seen that in both 2006 and 2017, most rural toilet retrofitting investment and farmers' medical and health expenditure in the provinces and cities are in the first and third quadrants, which further indicates that toilet retrofitting investment and its effects have significant spatial agglomeration characteristics. Therefore, when analyzing the influence of toilet retrofitting investment on farmers' medical and health expenditure, we cannot ignore the spatial correlation; otherwise, the estimation result will be biased.

### 4.2. Estimation results of all samples

The Hausman test results of the two weight matrices, support the selection of the fixed-effect model (FE). The results of the LR test and Wald test for spatial fixed effects (Table 3) also indicate that the LR value and Wald value are significant at the 1% level in the two weight matrices. $H_{0}^{1}$:θ = 0 and $H_{0}^{2}$:θ+ρβ = 0 are rejected, indicating that both spatial lag and spatial error exist, and the SDM cannot be simplified into SLM and SEM. Therefore, the SDM is selected.

**Table 3:** Estimation results of Spatial Durbin Model (SDM) under different weight matrices.

**Weight matrix**	**Spatial adjacency matrix**		**Geographic distance matrix**	
**Econometric model**	**FE**	**RE**	**FE**	**RE**
*RTI*	−0.055^***^ (0.014)	−0.047^**^ (0.020)	−0.038^***^ (0.010)	−0.032^***^ (0.008)
*PS*	−0.021^*^ (0.013)	−0.023^*^ (0.014)	−0.019^**^ (0.008)	−0.015^*^ (0.009)
*RH*	0.142^**^ (0.058)	0.116^***^ (0.031)	0.127^**^ (0.052)	0.082^**^ (0.033)
*PEF*	−0.230 (0.197)	−0.192 (0.166)	0.207 (0.375)	0.126^*^ (0.077)
*RMI*	0.107^***^ (0.028)	0.098^**^ (0.040)	0.092^**^ (0.037)	0.084^*^ (0.050)
*RLG*	0.034^**^ (0.013)	0.045^**^ (0.019)	0.040^***^ (0.011)	0.019^**^ (0.008)
*RPC*	0.056^**^ (0.024)	0.072^*^ (0.043)	0.063^**^ (0.026)	0.081^*^ (0.049)
W^*^RTI	0.023^***^ (0.006)	0.029^**^ (0.012)	0.025^***^ (0.007)	0.020^**^ (0.008)
W^*^PS	−0.029^**^ (0.012)	−0.024^**^ (0.010)	−0.031^**^ (0.013)	−0.028^*^ (0.007)
W^*^RH	0.218^**^ (0.089)	0.206^***^ (0.054)	−0.178^**^ (0.072)	−0.193^***^ (0.051)
W^*^PEF	−0.192^***^ (0.051)	−0.138^**^ (0.057)	0.154 (0.132)	0.0137 (0.116)
W^*^RMI	0.091^*^ (0.056)	0.057^**^ (0.023)	0.072^**^ (0.030)	0.065^***^ (0.017)
W^*^RLG	−0.045^**^ (0.018)	−0.063^***^ (0.016)	0.058^**^ (0.024)	0.074^**^ (0.030)
W^*^RPC	−0.036 (0.044)	−0.031 (0.039)	0.047 (0.058)	0.039 (0.052)
ρ	−0.493^***^ (0.127)	−0.451^***^ (0.118)	−0.624^***^ (0.163)	−0.572^***^ (0.151)
Sigma^2^	0.004^***^ (0.001)	0.001^***^ (0.000)	0.003^***^ (0.001)	0.002^***^ (0.000)
*R^2^*	0.893	0.824	0.925	0.906
Log-likelihood	385.072	406.238	431.752	415.028
Wald-err	46.58^***^	42.95^***^	50.31^***^	47.62^***^
Wald-lag	81.37^***^	73.52^***^	92.64^***^	79.48^***^
LR-err	54.19^***^	48.71^***^	60.28^***^	53.93^***^
LR-lag	62.64^***^	54.06^***^	70.59^***^	65.87^***^
Hausman test	22.75	*P* = 0.023	23.72	*P* = 0.000

*, ** and *** represent the significance level of 10, 5, and 1%, respectively, and the data in brackets is standard deviation.

Using Equation (3), the Maximum Likelihood method (ML) is employed to estimate the SDM under the spatial adjacency matrix and geographic distance matrix for all the samples (2006–2017), and the results are presented in Table 3. It is not difficult to find from Table 3 whether the maximum likelihood ratio under the geographic distance matrix is slightly higher than that under the spatial adjacency matrix, indicating that compared with the spatial adjacency matrix, the geographic distance matrix has a higher degree of fit. To further test the robustness of the model, this study compares and analyzes the regression results under two weight matrices—spatial adjacency matrix and geographic distance matrix. Under the SDM with spatial fixed effects, the ρ values of the two weight matrices are −0.493 and −0.624, which are both significant at the 1% level, indicating that there is an obvious spatial agglomeration effect in the influence of toilet retrofitting investment on farmers' medical and health expenditure, which is also consistent with the analysis of spatial correlation in the spatial correlation test. And the hypothesis 1 and hypothesis 2 are verified.

According to the partial differential method, the effect of toilet retrofitting investment on farmers' medical and health expenditure can be decomposed into total, direct and indirect effects, and the specific results are presented in Table 4.

(1) The influence of toilet retrofitting investment on farmers' medical and health expenditure.

**Table 4:** Effect decomposition of Spatial Durbin Model (SDM) under different weight matrices.

**Weight matrix**	**Spatial adjacency matrix**			**Geographic distance matrix**		
**Variables**	**Total effect**	**Direct effect**	**Indirect effect**	**Total effect**	**Direct effect**	**Indirect effect**
*RTI*	−0.045^**^ (0.019)	−0.033^**^ (0.014)	−0.012^*^ (0.007)	−0.057^***^ (0.015)	−0.032^***^ (0.008)	−0.015^**^ (0.006)
*PS*	−0.019^**^ (0.008)	−0.024^**^ (0.010)	0.005 (0.004)	−0.033^*^ (0.020)	−0.047^**^ (0.019)	0.014^*^ (0.008)
*RH*	0.097^*^ (0.058)	0.063^***^ (0.017)	0.034^***^ (0.009)	0.085 (0.103)	0.061^***^ (0.014)	0.024^**^ (0.010)
*PEF*	−0.180 (0.221)	−0.157 (0.196)	−0.023 (0.029)	−0.156 (0.194)	−0.183 (0.230)	0.027 (0.034)
*RMI*	−0.121^*^ (0.074)	−0.096^**^ (0.041)	−0.025^**^ (0.010)	−0.132^**^ (0.056)	−0.159^**^ (0.065)	0.027 (0.023)
*RLG*	0.023^**^ (0.010)	0.029^***^ (0.008)	−0.006 (0.005)	0.031^***^ (0.008)	0.023^**^ (0.010)	0.008^***^ (0.002)
*RPC*	0.027^***^ (0.007)	0.055^***^ (0.015)	−0.028^**^ (0.012)	0.041^**^ (0.017)	0.028^***^ (0.007)	−0.013^*^ (0.008)

*,** and *** represent the significance level of 10, 5, and 1%, respectively, and the data in brackets is standard deviation.

Table 4 indicates that the total effect of toilet retrofitting investment on farmers' medical and health expenditure is significantly negative, whether under the conditions of the spatial adjacency matrix or geographical distance matrix, but the total effect of the former is smaller than that of the latter. This indicates that toilet retrofitting investment can reduce farmers' medical and health expenditure, and the geographical distance factor can strengthen this effect. According to the decomposition results of spatial effects, when the spatial adjacency matrix and geographical distance matrix are employed, the direct effects of toilet retrofitting investment in this region are −0.033 and −0.032, and pass the significance test at the 5% and 1% level, respectively. This indicates that despite the type of spatial weight matrix used, toilet retrofitting investment has a significant direct effect on local farmers' medical and health expenditure. Under the spatial adjacency matrix and geographical distance matrix, the indirect effect of toilet retrofitting investment on the medical and health expenditure farmers in surrounding areas are −0.012 and −0.015, which pass the test at the 10% and 5% significance level, respectively. The indirect effect is negative, indicating that in the mechanism through which toilet retrofitting investment influences farmers' medical and health expenditure, there is a learning and imitating phenomenon among different subjects. Thus, when the rapid growth of toilet retrofitting investment in a province rapidly promotes local toilet retrofitting work, the advanced institution, experience and technology will be imitated by the government, communities, or enterprises, to improve the effect of toilet retrofitting and the physical health of local farmers in surrounding areas, thereby reducing farmers' medical and health expenditure (27). In general, under the setting of different weight matrices, the indirect effect of toilet retrofitting investment on farmers' medical and health expenditure is smaller than the direct effect. Thus, compared with the direct effect in the local area, the influence of toilet retrofitting investment on the medical and health expenditure of farmers in surrounding areas are small. Therefore, the hypothesis 2 is further verified.

(2) Other factors.

Table 3 indicates that under the setting of the two weight matrices, the direct effect of public service level on farmers' medical and health expenditure is significantly negative at the 5% level, indicating that improving public service level will reduce local farmers' medical and health expenditure. However, under the geographic distance matrix, the indirect effect of public service level only passes the significance test at the 10% level, it means that the geographical distance factor weakens the influence of public service on the medical and health expenditure of farmers in surrounding areas. Relevant research has confirmed that the higher level of public service contributes to the promotion of the surrounding governments to improve the investment in public services (37), and the increase of farmers' the medical and health expenditure in the surrounding areas.

The direct and indirect effects of rural medical and health level under the two weight matrices are significantly positive at the 1% and 5% levels. The direct effect is significantly positive, indicating that the extensive construction of rural clinics would promote local farmers' consumption of medical and health care, it is consistent with the research result of Dieleman et al. (38). The indirect effect is positive, indicating that the policies promoting the construction of rural clinics have achieved good results, and they are imitated by local governments, which accelerates the construction of rural clinics in neighboring areas, thereby increasing farmers' medical and health consumption.

Whether under the spatial adjacency matrix or geographical distance matrix, the direct and indirect effects of rural pension on farmers' medical and health expenditure are not significant. The possible explanation is that the elderly in rural society is mostly supported by their families (23), and the construction and popularization of nursing homes have not benefited the majority of rural elderly residents.

Considering both the spatial adjacency matrix and geographical distance matrix, the direct effect of rural medical insurance passes the significance test at the level of 5%, and the direct effect is negative, indicating that the higher the coverage rate of rural medical insurance, the lower farmers' medical and health expenditure. It has been confirmed that participation in medical insurance can significantly reduce the medical burden of rural residents (39). The indirect effect is negative only at the significance level of 5% under the setting of the spatial adjacency matrix, it means that the spatial adjacency factor will promote the surrounding governments encourage farmers to actively participate in the new rural cooperative medical system, thus increasing the farmers' medical and health expenditure in the surrounding areas.

The direct effect of rural minimum living guarantee under the two weight matrices passes the significance test at the 1% and 5% level, respectively, and the direct effect is positive, indicating that the fiscal expenditure of local governments on rural minimum living guarantee promotes farmers' medical and health expenditure. It is consistent with the research result of Yang and Gao (40), which suggest that subsistence allowance encourage rural households to invest on their medical and health. The indirect effect is positive at the 1% significance level under the geographical distance matrix, indicating that the geographical distance factor strengthens the influence of the rural minimum living guarantee on the medical and health expenditure of farmers in surrounding areas. With the increasing attention to the Poverty Alleviation Strategy and the continuous improvement of the social security system, the strategy of improving people's livelihood by increasing the financial expenditure on rural minimum living guarantee will be learned and imitated by the neighboring governments, to reduce farmers' medical and health expenditure in surrounding areas.

The direct effects of rural per capita consumption under the two weight matrix settings pass the significance test at the 1% level, and the direct effects are positive, indicating that improving rural residents' consumption level can promote them to increase their expenditure on medical and health care. Relevant literature has found that the higher consumption level in rural areas, the more local residents spend on healthy life, health products and medical service (41). The indirect effects are at the significance level of 5% and 10%, respectively; and indirect effect are negative, implying that the rapid increase in rural residents' consumption level will lead to the flow of various factors and resources to the area, make the supply of goods and services insufficient, and weaken the consumption expenditure of farmers in surrounding areas.

### 4.3. Intra-regional differences of the spatial effects of the influence of toilet retrofitting investment on farmers' medical and health expenditure

We divide the regions in China into eastern, central, western and northeast^④^, and analyze the total, direct and indirect effects of the toilet retrofitting investment in each region to explore the spatial effect of influence of toilet retrofitting investment on farmers' medical and health expenditure from the perspective of spatial characteristics. Spatial heterogeneity can reflect the differential effect of the influence of toilet retrofitting investment influence on farmers' health expenditure by the total, direct and indirect effects, respectively. Spatial correlation is closely related to the indirect effect, which indicates the influence of toilet retrofitting investment on the medical and health expenditure of farmers in surrounding areas. As the model estimation under the geographical distance weight matrix has a higher goodness of fit, the following will focus on the impact of toilet retrofitting investment on farmers' medical and health expenditure under the setting of geographical distance matrix. The estimation results are presented in Table 5.

**Table 5:** Estimated results of the subsamples of four regions 2006 to 2017.

**Region**	**Effect**	***RTI***	***PS***	***RH***	***PEF***	***RMI***	***RLG***	***RPC***
Eastern	Total effect	−0.038^**^ (0.016)	−0.023^***^ (0.006)	0.173^***^ (0.045)	−0.088^*^ (0.054)	−0.158^**^ (0.065)	0.030^**^ (0.012)	0.091^**^ (0.038)
	Direct effect	−0.025^***^ (0.007)	−0.016^***^ (0.004)	0.127^**^ (0.053)	−0.153 (0.135)	−0.102^**^ (0.042)	0.022^***^ (0.006)	0.063^***^ (0.016)
	Indirect effect	−0.013^*^ (0.008)	−0.007^**^ (0.003)	0.049 (0.042)	0.062 (0.054)	−0.056^*^ (0.033)	0.008^**^ (0.003)	0.028^***^ (0.007)
Central	Total effect	−0.049^***^ (0.013)	−0.043^***^ (0.011)	0.128^**^ (0.051)	−0.134^*^ (0.081)	0.174 (0.0154)	0.063^*^ (0.038)	0.059^**^ (0.025)
	Direct effect	−0.033^***^ (0.009)	−0.034^***^ (0.009)	0.095^***^ (0.025)	−0.176 (0.152)	−0.131^***^ (0.035)	0.047^***^ (0.013)	0.042^***^ (0.011)
	Indirect effect	−0.016^**^ (0.007)	−0.009^*^ (0.005)	0.033 (0.041)	0.042 (0.052)	−0.043^**^ (0.018)	0.016^*^ (0.010)	0.017^*^ (0.010)
Western	Total effect	−0.014^**^ (0.006)	−0.024 (0.021)	0.045^*^ (0.027)	−0.183 (0.156)	0.120^*^ (0.073)	−0.062^**^ (0.025)	0.018 (0.022)
	Direct effect	−0.024^**^ (0.010)	−0.030^*^ (0.018)	0.067^***^ (0.018)	−0.169 (0.145)	0.145^**^ (0.060)	−0.044^**^ (0.019)	0.031^***^ (0.008)
	Indirect effect	0.010^*^ (0.006)	0.006 (0.004)	−0.022^**^ (0.009)	−0.014 (0.017)	−0.025 (0.031)	−0.018 (0.023)	−0.013 (0.011)
Northeast	Total effect	−0.011 (0.013)	−0.024 (0.031)	−0.038^*^ (0.023)	−0.081 (0.069)	0.066^**^ (0.027)	−0.007^*^ (0.004)	0.026 (0.023)
	Direct effect	−0.018^**^ (0.007)	−0.020 (0.017)	−0.051^**^ (0.022)	−0.093 (0.113)	0.084^***^ (0.022)	−0.009 (0.008)	0.035^*^ (0.021)
	Indirect effect	0.007 (0.009)	−0.004 (0.003)	0.013 (0.016)	0.012 (0.015)	−0.018 (0.016)	0.002 (0.003)	−0.009 (0.008)

*, **, and *** represent the significance level of 10, 5, and 1%, respectively, and the data in brackets is standard deviation.

The estimated results in Table 5 reveal the following:

(1) Generally, an increase in rural toilet retrofitting investment will reduce the farmers' medical and health expenditure in most areas. The improvement of rural health care, rural medical insurance and the minimum living guarantee and per capita consumption level will increase farmers' medical and health expenditure in most areas. Because the level of public service input varies greatly in different regions, the effect of public service input would be different in different regions. However, the level of old-age care in rural areas has no significant impact on farmers' medical and health expenditure.

(2) From the comparison of the four regions, in terms of spatial heterogeneity, toilet retrofitting investment reduces the medical and health expenditure of farmers in most regions of China. The total effect is in the following order: central > eastern > western, but that of the northeast is not significant; the effect value of direct effect is in the following order: central > eastern > western > northeast. The indirect effect is significantly negative in eastern and central, significantly positive in western, but not significant in northeast. From the perspective of spatial correlation, in the eastern and central regions, toilet retrofitting investment can reduce the medical and health expenditure of farmers in surrounding areas, indicating that the investment drive has an obvious “demonstration effect” among provinces. Thus, when a province in the eastern or central region increases the toilet retrofitting investment to improve farmers' health and quickly achieves a significant result, surrounding areas will follow suit. In the western region, the toilet retrofitting investment can increase the medical and health expenditure of farmers in the surrounding areas, indicating that increasing toilet retrofitting investment will lead to a competition effect. Thus, the demand for toilet retrofitting caused by investment in a western province will trigger fierce competition in related industries and factor markets, leading to the flow of factors from surrounding provinces to the province, which will hinder toilet retrofitting work in surrounding areas and make it difficult to effectively improve the rural residents' health in surrounding areas. This is consistent with the research result of Gao et al. (18). Toilet retrofitting investment in the northeast does not have a significant influence among provinces. It may be because the relatively weak financial capacity of the government and cold climate leads to a limited effect of the rural toilet retrofitting investment in these areas (42), thus limiting its influence on the medical and health expenditure of farmers in surrounding areas. And the hypothesis 3 is verified.

### 4.4. Inter-regional differences of the spatial effects of the influence of the toilet retrofitting investment on farmers' medical and health expenditure

To explore the spatial characteristics of toilet retrofitting investment and its health effects, the influence of toilet retrofitting investment on farmers' medical and health expenditure in different regions will be further analyzed. Based on the above, the weight matrix is adjusted accordingly by referring to the treatment methods of spatial correlation in different groups, so that the spatial correlation between rural toilet retrofitting investment and its health effects among different regions can be compared in pairs. For specific treatment methods, refer to the studies of Ledyaeva (43) and Zhong (36). The estimated result of the spatial effect of toilet retrofitting investment on farmers' medical and health expenditure among the four regions is presented in Table 6.

**Table 6:** Estimation results among four regions from 2006 to 2017.

**Explanatory variable**	**Eastern–Central**	**Eastern–Western**	**Eastern–Northeast**	**Central–Western**	**Central–Northeast**	**Weastern–Northeast**
*RTI*	−0.012^**^ (0.005)	−0.009 (0.011)	−0.006^*^ (0.004)	−0.027^***^ (0.007)	−0.013^*^ (0.008)	−0.015^**^ (0.006)
P	0.471^***^ (0.122)	−0.395^***^ (0.105)	−0.552^***^ (0.146)	−0.328^***^ (0.086)	−0.416^***^ (0.109)	0.183^***^ (0.048)
Sigma^2^	0.022^***^ (0.006)	0.007^***^ (0.002)	0.024^***^ (0.006)	0.016^***^ (0.004)	0.015^***^ (0.004)	0.009^***^ (0.002)

*, **, and *** represent the significance level of 10, 5, and 1%, respectively, and the data in brackets is standard deviation.

Table 6 shows that the spillover effects of rural toilet retrofitting investment in eastern–central, eastern–western, eastern–northeast, western–western, western–northeast and western–northeast regions are−0.012,−0.009,−0.006,−0.027,−0.013, and−0.015, respectively. The intensity of the regional interaction between the central and western regions is the greatest, followed by that of western–northeast, central–northeast and eastern–central, the intensity of the eastern–northeast is least; the influence in eastern–western is not significant. Due to their proximity, similar natural geographical environment and social-economic development level, the governments have focused on supporting toilets retrofitting in the central and western regions in recent years (7), which has an implication on the promotion of rural toilet retrofitting work to realize technology diffusion and experience reference and institutional imitation. Therefore, its regional influence is the strongest. Due to the geographical distance and the gap in the social and economic development of the regions, the interaction in central–northeast and eastern–central is weak. The interaction between east and northeast is the least because of the great difference in natural geographical environment. The correlation in the eastern–western region is not significant. A possible reason is that local public service, rural health care, minimum living guarantee and rural per capita consumption are different among the regions. Therefore, the investment-driven rural toilet retrofitting cannot affect surrounding areas in a short time. And the hypothesis 3 is further verified.

### 4.5. Limitations and perspectives

Several limitations exist in this research. First, limited by the data availability, the panel data we used is only at the province level. Future research will use micro-survey data among different regions to enhance the representativeness and persuasiveness of research result. Second, as an important part of rural human settlement environment, toilet retrofitting can not only improve the health and wellbeing of residents, but also change the village appearance, enhance the village attractiveness, and attract talents return to build the countryside. The resource allocation effect of rural toilet retrofitting is a consideration to explore in the future work.

## 5. Conclusions

Based on the above analysis, this study uses the panel data of 30 provinces in China from 2006 to 2017 and employs the spatial econometric model, to investigate the spatial effect of toilet retrofitting investment on farmers' medical and health expenditure. It draws the following conclusions:

1. From 2006 to 2017, there were significant spatial agglomeration characteristics of toilet retrofitting investment and farmers' medical and health expenditure. Therefore, when exploring the spatial effect of the influence of toilet retrofitting investment on farmers' medical and health expenditure, the spatial heterogeneity and spatial correlation between them should not be ignored; otherwise, the estimation results would be biased.
2. At the national level, under the geographical distance matrix, the total, direct and indirect effects of rural toilet retrofitting investment on farmers' medical and health expenditure are all negative, and the direct effect is greater than the indirect effect.
3. For the comparison within different regions, in terms of spatial heterogeneity, the total effect of toilet retrofitting investment is in the following order: central > eastern > western, and that of the northeast is not significant. The order of the direct effect is central > eastern > western > northeast. The indirect effect is significantly negative in the eastern and central regions, positive in the western region, and insignificant in the northeast region. From the perspective of spatial correlation, the investment driven rural toilet retrofitting in the eastern and central regions will cause surrounding regions to follow, leading to a spillover effect of toilet retrofitting investment. Toilet retrofitting investment in the western regions will lead to fierce competition in relevant industries and factor markets, which is manifested as the competition effect.
4. From the perspective of the interaction between regions, toilet retrofitting investment has spillover effects among four regions, and the intensity of the effect between the central and western regions is the largest, followed by is western–northeast, central–northeast and eastern–central, the intensity of eastern–northeast is least. The effect between eastern–western is not significant.

Based on the above conclusions, the following policy implications are presented: First, the direct effect of rural toilet retrofitting investment on farmers' medical and health expenditure is much greater than the indirect effect on surrounding areas. Therefore, in the overall planning of the regional rural toilet retrofitting work, it is more important to accurately understand the local climate and geographical characteristics as well as economic and social conditions and learn from the toilet retrofitting technology and management mode to improve the efficiency of toilet retrofitting investment. Second, the comprehensive promotion of rural toilet retrofitting by the Chinese government has improved farmers' physical health status, but the phenomenon of regional imbalance in rural toilet retrofitting has not improved radically. Therefore, the central government needs to invest more resources in poor economic environments and high cold and dry areas to ensure that these regions can overcome various constraints and realize the widespread usage of rural sanitary toilets. Finally, regions with close geographical distance often have similar physical as well as geographical conditions and economic and social environments, which is conducive to the spillover effect between them. Therefore, western underdeveloped regions should learn the toilet retrofit technology and management mode from developed areas and appropriately strengthen communication and cooperation with neighboring regions. It would play a positive role in institutional imitation among local governments, which will lead to a “learning effect” among enterprises and communities and promote local toilets retrofitting through “learn by seeing” and “learn by doing” to improve farmers' health. In addition, it is necessary to strengthen the research and development of toilet retrofitting technology in alpine and arid areas, strengthen the supply of public services, improve social security system, increase farmers' income, and consider local characteristics; this would lead to a spillover effect, and reduce farmers' medical and health expenditure.

## Data availability statement

The raw data supporting the conclusions of this article will be made available by the authors, without undue reservation.

## Author contributions

XZ conceived and designed the paper, collected, analyzed and interpreted the data, and wrote the paper. F-XY and D-SF analyzed and interpreted the data and took constructive advices for the article writing. Z-NY collected and analyzed the data. All authors contributed to the article and approved the submitted version.

## Appendix

### Annotation

① The data comes from http://www.gov.cn/xinwen/2021-04/08/content_5598294.htm?gxj.
② According to “China Rural Statistical Yearbook,” the per capita living consumption expenditure of rural resident contains food expenditure, clothing expenditure, housing expenditure, household equipment and supplies and services expenditure, transportation and communication expenditure, cultural, educational and entertainment supplies and services expenditure, medical and health expenditure and other expenditure. Medical and health expenditure is the expenditure per capita of rural households on medical and health drugs, medical equipment and health services.
③ The abbreviation of Chinese 30 provinces: BJ, Beijing; TJ, Tianjin; HE, Hebei; SX, Shanxi; IM, Inner Mongoria; LN, Liaoning; JL, Jilin; HL, Heilongjiang; SH, Shanghai; JS, Jiangsu; ZJ, Zhejiang; AH, Anhui; FJ, Fujian; JX, Jiangxi; SD, Shandong; HA, Henan; HB, Hube; HN, Hunan; GD, Guangdong; GX, Guangxi; HI, Hainan; CQ, Chongqing; SC, Sicuan; GZ, Guizhou; YN, Yunnan; SN, Shaanxi; GS, Gansu; QH, Qinghai; NX, Ningxia; XJ, Xinjiang.
④ China is divided into four regions: the eastern region includes Beijing, Tianjin, Hebei, Shandong, Jiangsu, Fujian, Guangdong, Shanghai, Zhejiang and Hainan; the central region includes Shanxi, Anhui, Henan, Hubei, Hunan and Jiangxi; the western region includes: Inner Mongolia, Xinjiang, Qinghai, Gansu, Shaanxi, Chongqing, Ningxia, Sichuan, Guizhou, Yunnan and Guangxi; The northeast includes Liaoning, Jilin and Heilongjiang; The Chinese territories of Tibet, Hong Kong, Macau and Taiwan are not included.
